# Cerebellovascular Disease: Magnetic Resonance Imaging

**DOI:** 10.5334/jbr-btr.1230

**Published:** 2016-11-19

**Authors:** Laurens Jaak De Cocker

**Affiliations:** 1 Kliniek Sint-Jan, Brussels, Belgium; 2 UMC Utrecht, Utrecht, the Netherlands

**Keywords:** cerebellum, MRI, Arterial Spin Labeling, 7-Tesla MRI, PICA, infarct

## Abstract

The goal of this thesis was to elucidate the details of cerebellovascular diseases with advanced magnetic resonance (MR) imaging (MRI) and to translate the findings to routine clinical MRI. The first aim was to image cerebellar arterial perfusion territories, which was achieved by applying super-selective arterial spin labelling (ASL) MRI with labelling of both vertebral arteries in addition to the carotid arteries. The second aim was to unravel the imaging patterns of cerebellar infarctions with 7T post-mortem MRI in addition to volume (3D) clinical MRI. This research led to the description of “cerebellar cortical infarct cavities”, an incidental imaging finding that proved to be the most frequent manifestation of cerebellar ischemia as well as a marker of atherosclerotic and thromboembolic cerebrovascular disease. Finally, we found that almost all patients with such cavities lack a clinical history of vertebrobasilar transient ischemic attack (TIA) or stroke, disclosing the still very high incidence of clinically occult ischemia in the posterior fossa.

## Introduction

The goal of this thesis was to elucidate the details of cerebellovascular diseases with advanced magnetic resonance (MR) imaging (MRI) and to translate the findings to routine clinical MRI. More specifically, the aims of this thesis were to image cerebellar perfusion territories, to precisely describe the imaging patterns of cerebellar infarctions, and to investigate the commonly observed incidental cerebellar cavities on MRI. This pictorial essay gives an illustrated overview of the highlights of this thesis with the objective to increase the diagnostic potential of MRI for cerebellar infarctions in clinical practice.

## Imaging of Cerebellar Perfusion Territories with ASL-MRI

The first aim of this thesis was to critically appraise and improve the classification of small cerebellar infarctions and to visualise arterial cerebellar perfusion territories. Although small cerebellar infarcts are traditionally classified into “watershed” or “border zone” perfusion territories, arterial perfusion territories and the border zones in between them are widely variable among subjects. Also, many infarcts do not fit into such a classification system, which hinders its use in clinical practice [1].

We proposed two answers to these limitations. The first was to omit the traditional classification and to classify small cerebellar infarctions according to anatomical location in the cerebellum instead of arterial perfusion territories [1]. The second and more challenging answer was to develop the first imaging technique to visualise cerebellar perfusion territories in vivo [2]. This way, cerebellar infarction may be directly linked with the responsible diseased artery, for instance, to define whether a stenosis should be considered symptomatic.

**Figure 1:**
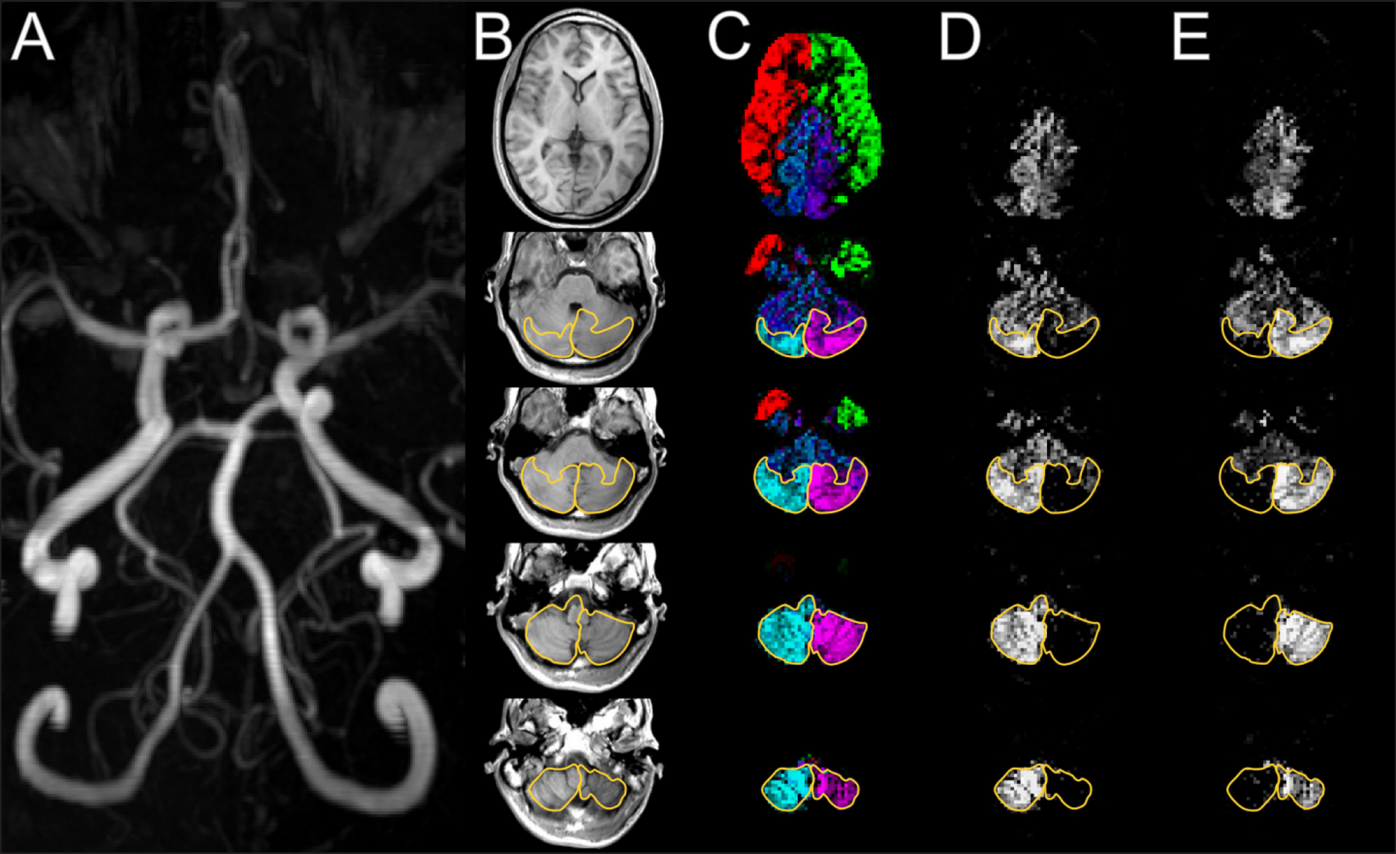
Time-of-flight MRA **(A)** shows a slightly dominant left vertebral artery. The anatomical T1-weighted images **(B)** show through the cerebellum from inferior (bottom row) to superior (top row). Territorial perfusion images **(C)** show the territory supplied by the right (cyan) and left (magenta) vertebral arteries and by the right (red) and left (green) internal carotid arteries. With the mixing of blood in the basilar artery, cyan and magenta turn into blue. Individual perfusion images of the right **(D)** and left **(E)** vertebral arteries. The posterior inferior cerebellar artery (PICA) territories are outlined in perfusion images **(C–E)** and copied to the anatomical images **(B)**. The PICA territories can be easily discriminated; they are symmetrical and supply the posterior cerebellar surfaces **(B)**. Reproduced from *Neuroimage* [2].

**Figure 2:**
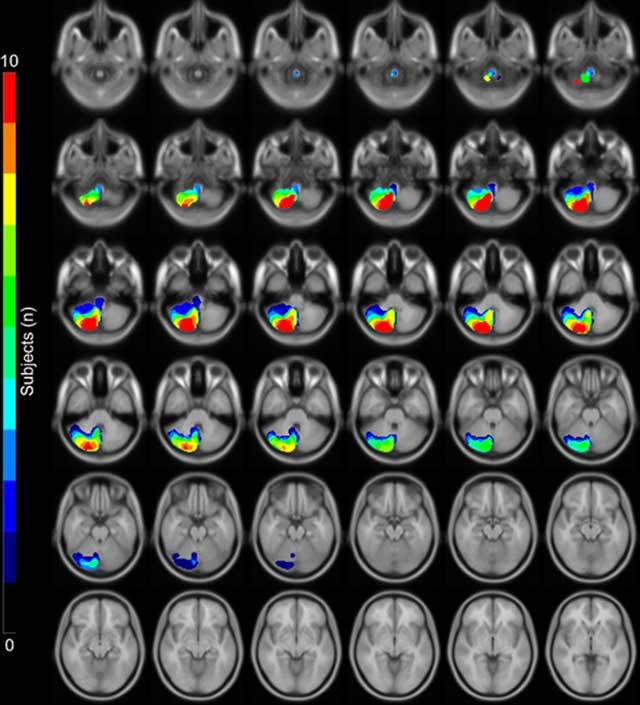
This is a statistical map of the extent of the perfusion territories of the right posterior inferior cerebellar arteries (PICA) as visualized in 10 healthy subjects with super-selective ASL superimposed on a standard MNI space template. The depicted images are orientated axially from inferior (top row) to superior (bottom row) [2].

To visualise cerebellar perfusion territories, we studied healthy subjects with super-selective ASL-MRI [2,3,4]. ASL-MRI is a non-invasive technique to measure brain perfusion without the need to administer an exogenous contrast agent. In ASL, arterial blood water is magnetically labelled and then imaged. By labelling both vertebral arteries in addition to both internal carotid arteries, we were able to distinguish the territories supplied by one vertebral artery (posterior inferior cerebellar artery/PICA) from those supplied by both vertebral arteries (anterior inferior cerebellar artery/AICA and superior cerebellar artery/SCA) and from the contralateral vertebral artery (contralateral PICA) (Figure 1) [2]. Since the PICA is the only artery originating from the vertebral artery, the ASL signal within the PICA territory is only observed with labelling of the ipsilateral vertebral artery, while labelling of the contralateral vertebral artery results in a signal void. Of note is that the technique only allows separation of one of the three cerebellar perfusion territories, and this territory corresponds to the PICA territory in regular anatomy. Nevertheless, variant anatomy is common; for example, the AICA can also stem from the vertebral artery, such as in the case of a common PICA-AICA trunk. Interpretation of the ASL dataset therefore needs close correlation with arterial anatomy, usually by MR angiography (MRA). Application of the described technique allowed us to create a probabilistic flow territory map of the PICA-perfusion territory based on 10 healthy subjects (Figure 2) [2].

## Imaging Patterns of Cerebellar Infarctions

**Figure 3:**
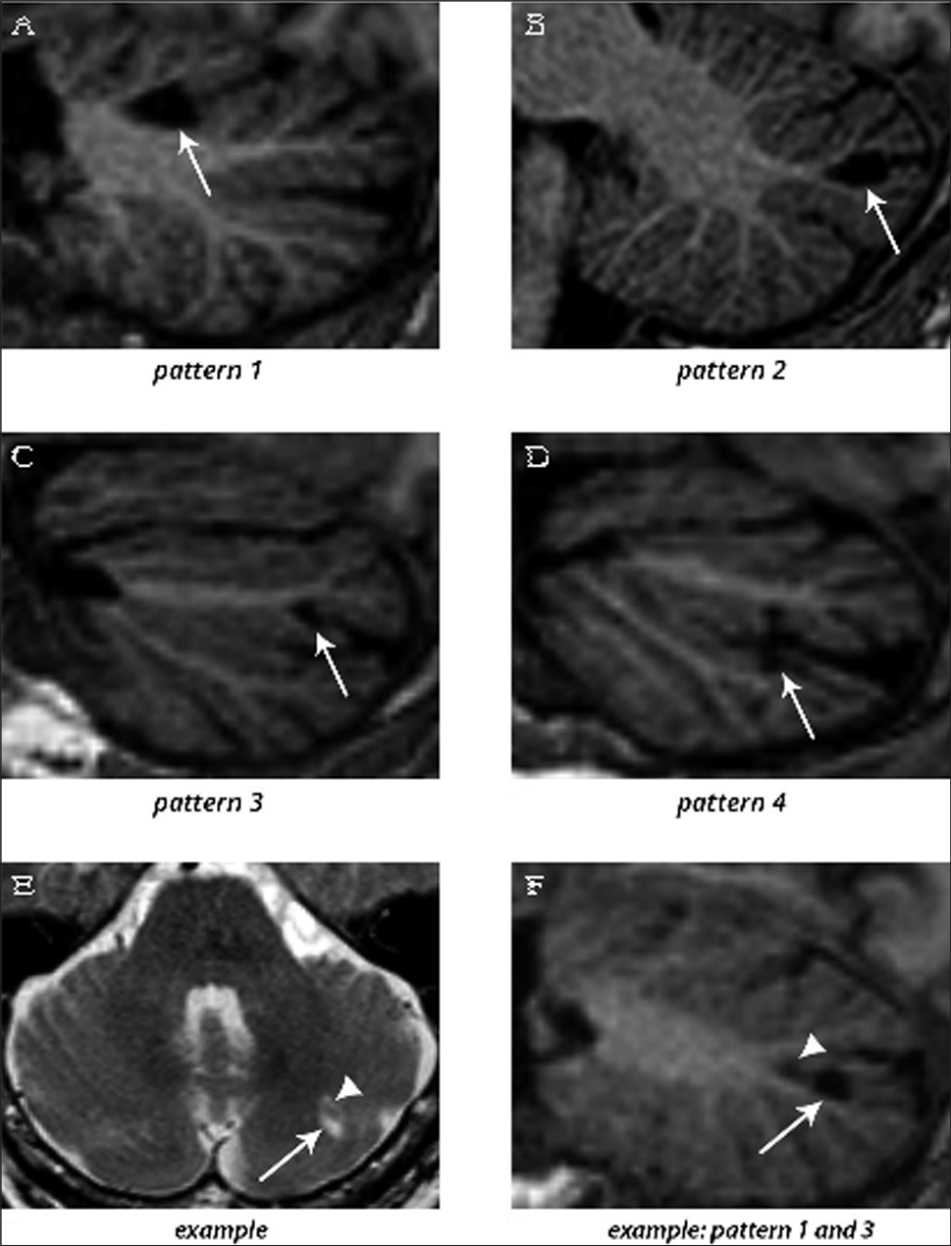
3D T1-weighted images (sagittal reconstructions) of the brain **(A–D** and **F)**, providing excellent contrast between gray matter and white matter, show the four patterns by which cerebellar infarcts (arrows) typically affect the cerebellar cortex. An example of two cerebellar infarcts adjacent to each other (**E**; arrow and arrowhead) are detected on transverse T2WI. Sagittal reconstruction of 3D T1WI of the same two infarcts **(F)** shows how the topography of one infarct corresponds to pattern 3 (arrow), while the other infarct (arrowhead) corresponds to a small pattern 1 infarct. Notice the sparing of the subcortical and deep white matter in each infarct. The images are cropped to only display the cerebellum. Reproduced from *Neuroimage: Clinical* [5].

The second aim of this thesis was to map the precise MRI patterns of cerebellar infarcts. Thus far, imaging studies of cerebellar infarctions have been grossly restricted to acute infarcts, with routine axial imaging providing limited contrast between gray and white matter. Nevertheless, many cerebellar infarcts present as an incidental finding beyond the diffusion-weighted-imaging (DWI) positive acute stage, and there is an ongoing evolution of brain MRI protocols from 2D (usually axial) towards 3D (volume) acquisitions. Therefore, we investigated the MRI patterns of incidental cerebellar infarcts in 3D, including an assessment of gray and white matter involvement, with volume acquisitions on 1.5T MRI [5]. This way, we found that cerebellar infarcts show a remarkable affinity for the cerebellar cortex (Figure 3) [5]. Small cerebellar infarcts involved the cerebellar cortex in isolation, and large cerebellar infarcts involved the cerebellar cortex in combination with a variable degree of subjacent white matter. But no infarcts involved subcortical branches of white matter in isolation [5]. We also found that, unlike cerebellar infarcts studied in the symptomatic stage, 90 percent of cerebellar infarcts measured less than 1 cm [5]. Finally, almost all infarctions showed evidence of cavitation, which renders them very conspicuous on MRI due to a high intrinsic contrast between intracavitary fluid and surrounding tissues. For the reasons above, we introduced the term “cerebellar cortical infarct cavity” to denote a small infarct cavity in the cerebellar cortex. These cerebellar cortical infarct cavities accounted for the overwhelming majority of incidentally found cerebellar infarctions.

## Cerebellar Cortical Infarct Cavities

**Figure 4:**
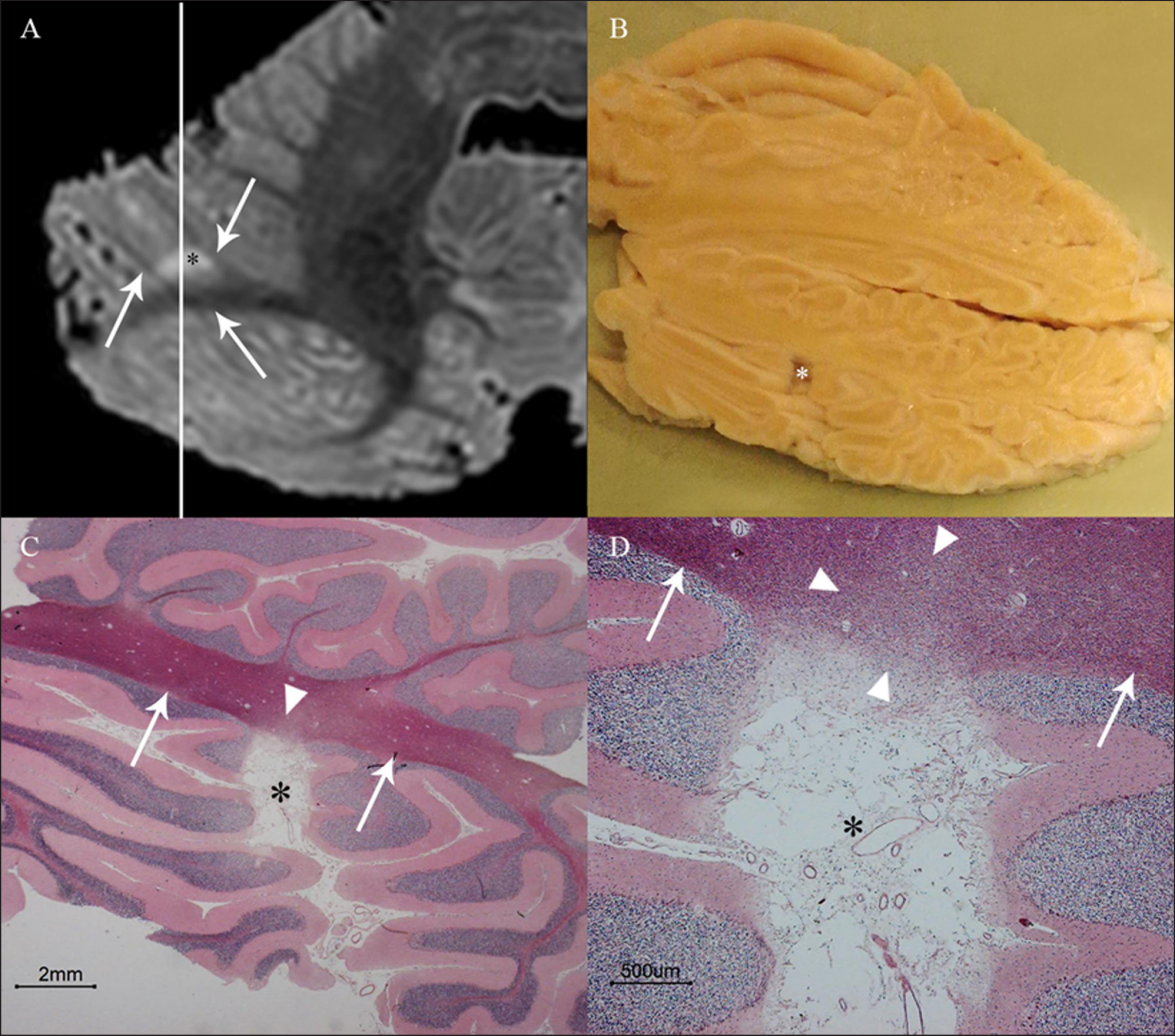
This image shows cerebellar cortical infarct cavity (asterisk) in the right cerebellar hemisphere on post-mortem 7T MRI (**A**; axial T2WI), gross specimen cut along the line indicated in A **(B)**, and microscopy (**C–D**; HE stain). Notice the destruction of all three cortical layers on microscopy **(C–D)** and a preserved juxtacortical white matter (arrows in **A, C**, and **D**) with some microscopic gliotic changes (arrowheads in **C** and **D**). The surroundings of the cerebellum are dark on T2WI because the specimens were submerged in Fomblin **(A)**, which does not yield MRI signal. Reproduced from *Cerebrovascular Diseases* [6].

**Figure 5:**
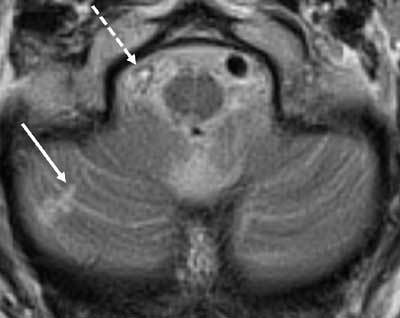
This image shows a small cortical infarct (arrow) in the posterior lobe of the right cerebellar hemisphere related to a thrombosed right vertebral artery (dashed arrow).

The third and last aim of this thesis was to investigate cerebellar cortical infarct cavities as a potential marker of cerebrovascular disease. First, we performed a radiologic-pathologic post-mortem validation study in which we examined 40 whole cerebella with 7T MRI and histopathology (Figure 4) [6]. All cavities retrieved on histopathological examination proved to be compatible with cavities of ischemic origin. Moreover, the study confirmed that the vast majority (20/22) of cerebellar infarct cavities occur in the cortex with sparing of subjacent white matter, except for some microscopic white matter extension exclusively visible on histopathological specimens [6]. Second, we performed an epidemiological study of 636 patients with vascular disease, and we correlated cerebellar cortical infarct cavities on 1.5T MRI with vascular risk factors, MRI markers of cerebrovascular disease, and mental and physical functioning [7]. In this study, we found cerebellar cortical infarct cavities in 10 percent of patients with vascular disease and mean patient age of 62 years, suggesting that cerebellar infarcts in general are far more common than may have been previously assumed based on symptomatic case series [7]. In addition, we found a significant relationship of cerebellar cortical cavities with markers of atherosclerosis, thromboembolic cerebrovascular disease and worse physical functioning (Figure 5) [7].

Identification of these cavities increases the visible burden of brain infarcts on MRI, and recent studies indicate that the total burden of (larger and smaller) infarcts may explain patients’ (functional) status, including cognitive performance and prognosis [7]. Also, cerebellar cortical infarct cavities add insights to the temporal and spatial (territorial) distribution of brain infarcts in individual patients [7]. In case of cerebellar cortical infarct cavities, patient’s cerebrovascular risk factors should be evaluated and optimisation of preventive therapy be considered [7].

Third and finally, we established the relationship of cerebellar cortical infarct cavities and symptomatic vertebrobasilar ischemia with prior history of vertebrobasilar transient ischemic attack (TIA) or stroke [8]. We evaluated 46 patients with a recent vertebrobasilar TIA or stroke and a symptomatic vertebral artery stenosis greater than 50 percent [8]. In this study, cerebellar cortical infarct cavities proved to be strongly associated with vertebral artery disease, as the cavities were observed in one-third of patients with symptomatic vertebral artery disease as opposed to 10 percent of patients of similar age with arterial disease in general [7]. Despite their ischemic origin, only 1 of 16 patients with cerebellar cortical infarct cavities was known with a prior history of vertebrobasilar TIA or stroke, indicating the very high percentage of occult ischemic events in the cerebellum [8]. As widely known, cerebellar infarcts may be clinically misdiagnosed because of non-specific clinical findings, such as nausea and dizziness, or simply may be not listened to due to lack of awareness of stroke-like symptoms in the elderly and their relatives.

In addition, acute cerebellar infarcts are easily missed on CT scans. Even so important, because of availability issues, MRI scans are often only performed beyond the acute stage of diffusion restriction. Since infarcts may undergo “fogging” in the subacute stage on both CT and MRI, many recent cerebellar infarcts are missed not only on clinical grounds but on radiological grounds as well. Afterwards, however, these infarcts become very conspicuous again on MRI due to cavitation [5,6]. All of the above may account for the frequent discovery of incidental cerebellar infarctions on MRI.

## Conclusion

In conclusion, the work in the present thesis introduced the visualisation of cerebellar arterial perfusion territories in vivo with super-selective ASL-MRI, which may allow for a more precise assessment of cerebellar infarctions in the future. Despite all medical and technical advancements, most cerebellar infarcts remain unnoticed initially and are instead only detected as an incidental cortical cavity later in life. Similar to small cortical infarcts in the cerebrum, cerebellar cortical infarcts are a marker of atherosclerotic and thromboembolic cerebrovascular disease.

This work was submitted and presented in fulfillment of the requirements for the degree of philosophical doctor in Utrecht on December 10, 2015.
